# Climate factors determine the utilization strategy of forest plant resources at large scales

**DOI:** 10.3389/fpls.2022.990441

**Published:** 2022-08-10

**Authors:** Jiangfeng Wang, Xianxian Wang, Yuhui Ji, Jie Gao

**Affiliations:** ^1^College of Life Sciences, Xinjiang Normal University, Ürümqi, China; ^2^Key Laboratory of Earth Surface Processes of Ministry of Education, College of Urban and Environmental Sciences, Institute of Ecology, Peking University, Beijing, China

**Keywords:** resource utilization strategies, vertical levels of communities, climatic factors, soil nutrient factors, macroscale

## Abstract

Plant functional traits are a representation of plant resource utilization strategies. Plants with higher specific leaf area (SLA) and lower leaf dry matter content (LDMC) exhibit faster investment-return resource utilization strategies. However, the distribution patterns and driving factors of plant resource utilization strategies at the macroscale are rarely studied. We investigated the relative importance of climatic and soil factors in shaping plant resource utilization strategies at different life forms in forests using data collected from 926 plots across 163 forests in China. SLA and LDMC of plants at different life forms (i.e., trees, shrubs, and herbs) differ significantly. Resource utilization strategies show significant geographical differences, with vegetation in the western arid regions adopting a slower investment-return survival strategy and vegetation in warmer and wetter areas adopting a faster investment-return survival strategy. SLA decreases significantly with increased temperature and reduced rainfall, and vegetation growing in these conditions exhibits conservative resource utilization. Mean annual precipitation (MAP) is a key climatic factor that controls the resource utilization strategies of plants at the macroscale. Plants use resources more conservatively as soil pH increases. The influence of climate and soil factors is coupled to determine the resource utilization strategies of plants occupying different life forms at the macroscale, but the relative contribution of each varies across life forms. Our findings provide a theoretical framework for understanding the potential impact of increasing global temperatures on plant resource utilization.

## Introduction

Plant functional traits are physiological and morphological characteristics of plants that respond to environmental changes, significantly affecting the function of forest ecosystems (Cappelli et al., 2020; Li X. et al., 2021; Weemstra et al., 2021). Specific leaf area (SLA) and leaf dry matter content (LDMC) are significantly correlated with particular plant resource utilization strategies and are extremely sensitive to climate change (Kattge et al., 2020). SLA is the leaf area per unit of dry leaf mass (Worthy et al., 2020) which, to some extent, reflects the ability of plant leaves to capture light energy under different environmental conditions. SLA also reflects the survival strategy adopted by plants to maximize carbon capture and is positively correlated with potential growth rate and maximum photosynthetic rate (Liu et al., 2018). Plants living in barren, high-temperature, and arid environments allocate more resources to enhance the dry matter content of foliage, which buttresses leaves against adverse conditions. At the same time, plants growing in these environments reduce SLA and photosynthetic rate to prolong leaf lifespan (Dwyer et al., 2014; Gong and Gao, 2019). LDMC is the ratio of leaf dry weight to fresh weight, which measures vegetation’s response to aridity and is closely related to the leaf water holding capacity (Smart et al., 2017). Smaller cell gaps and higher diffusion resistance in plants reduce leaf transpiration and total respiration, resulting in increased LDMC (Baribault et al., 2010). Studies have shown that plants with high SLA and low LDMC access resources more easily (Siefert and Ritchie, 2016), allowing for a faster investment-return survival strategy.

Plants adapt to local environmental conditions by modulating leaf functional traits to regulate their capacity to assimilate carbon and obtain nutrients (Yin et al., 2018). Mean annual precipitation (MAP) and mean annual temperature are the climate factors with the greatest impact on SLA (He et al., 2010), and both are strongly positively correlated to SLA (Firn et al., 2019). When rainfall is low, plant LDMC increases, reflecting vegetation’s investment in leaf construction to improve survival odds (Jiang et al., 2017). Increases in precipitation result in enrichment of the soil solution in dissolved salts, which changes soil conductivity. Overall, increased precipitation supports plant growth due to its association with increased SLA, transpiration rate, and net photosynthetic rate and a decrease in LDMC (Pietsch et al., 2014; Gong and Gao, 2019). Plants with high LDMC conserve water more efficiently due to lower rates of transpiration (Suter and Edwards, 2013). Temperature and precipitation jointly determine the resource acquisition and utilization trade-offs of plant leaves. Within a given forest community, differences in vegetation type (i.e., tree, shrub, or herb) and nutrient uptake strategies make it difficult to determine the resource utilization strategies employed by different groups (Poorter et al., 2009). Therefore, it is particularly important to quantify the macroscale effects of climate factors on SLA and LDMC of plants occupying different life forms in forests.

The availability of soil nutrients influences spatiotemporal patterns of leaf functional traits as well as plant resource utilization (Newsham et al., 2016; Huang et al., 2021). Total nitrogen content and the ratio of carbon to nitrogen are positively correlated with SLA and negatively correlated with LDMC (Hodgson et al., 2011). The effects of N limitation on plant growth are mitigated in soils enriched in N, thereby increasing SLA and LDMC (Wang et al., 2022). The availability of phosphorus in the soil is pH-dependent and, under acidic conditions, plants can rapidly access P to maximize growth. They also tend to have higher SLA and lower LDMC (Tao et al., 2019). Though soil nutrients have an effect on plant growth, the main drivers affecting leaf resource utilization strategies have not yet been identified (Cochrane et al., 2016; Poorter et al., 2019).

Leaf functional traits indicate the survival strategies of plants under various environmental conditions (Li X. et al., 2021). Environmental variation is also reflected in the trade-offs between plant access and utilization of resources, with significant differences in resource utilization strategies appearing across environmental gradients (Storkey and Macdonald, 2022). Characteristics of community system diversity make community trait variation a more effective indicator of plant responses to environmental change than species traits alone (Poorter et al., 2009), which ignore interspecific interactions (Taylor et al., 2015). In general, community functional traits are a better indicator of the ability of plants to access resources than the traits of individual species (Liu et al., 2021). At the community level, the resource utilization strategies of plants occupying different life forms are significantly different (Cheng et al., 2022). When shrubs and herbs are shaded, they adopt a faster investment-return survival strategy by increasing SLA to produce more organic matter through more efficient light interception and enhanced photosynthetic rates (Rüger et al., 2012). In contrast, if the leaves of trees receive significant sunlight, intense respiration drives dry matter depletion, resulting in reduced LDMC. When exposed to continuous light, plants must decrease SLA to lower synthetic rate in order to minimize damage caused by solar radiation (Li Y. et al., 2021). SLA reduction is a survival strategy that allows plants to better capture light energy and increase LDMC to prolong leaf lifespan (Liu et al., 2018).

Numerous studies have found that plant resource utilization strategies are complex due to their regulation by a combination of environmental factors (Huang et al., 2021; Wang et al., 2022). Exploring the distribution patterns of key leaf functional traits at large scales is of great ecological importance to quantify the effects of environmental conditions on plant resource utilization strategies (Li et al., 2022). Based on field survey data from 163 sites in China, we sought to identify the key drivers affecting leaf resource utilization strategies at different vegetative levels in forest communities. We propose the following hypotheses: (1) at the macroscale, plant resource acquisition strategies vary between life forms; (2) average annual precipitation is the dominant factor affecting the resource utilization strategy of plants at different life forms; and (3) climatic conditions are the dominant environmental factors affecting leaf resource acquisition strategies at different life forms.

## Materials and methods

### Study area and sample data

China has a vast territory and diverse climate, with 23% of its area covered by a rich and diverse array of forest ecosystem types. Using 926 forest plots from 163 sites in China surveyed between 2005 and 2020 (Supplementary Figure 1), we explored the spatial distribution patterns and driving factors driving resource utilization strategies at different life forms of forest communities. Forest communities are divided into tree, shrub, and herb levels according to species composition, structure, and production, with each plant having its life form, and vertical differentiation provides a good indication of the community’s adaptation to environmental conditions (Latham et al., 1998). We randomly selected at least four representative sample plots at each site and recorded the longitude, latitude, and altitude of each plot. Our study sites ranged from 19.1°N to 53.5°N, from 79.7°E to 129.3°E, and altitudes ranged from 1 to 4,000 m.

### Functional data

Leaf traits are widely used to indicate the adaptation of plants to their environment (Yu et al., 2022). The functional traits selected were SLA and LDMC, which are closely related to light interception, resource utilization, growth, and development of plants (Brun et al., 2022).

The experiment used the Japanese Cano Scan LIDE 110 portable leaf area meter to measure the area of fresh single leaf leaves after petiole removal. The fresh leaf weight was measured by electronic balance (accuracy of 0.0001 g), and then the fresh leaves were placed in a drying box at 105°C for 15 min, after which the temperature was reduced to 60°C for 48 h. The leaf dry weight was measured by weighing with a 1/10,000 electronic balance after drying at (Nanes, 2015). SLA (m^2^/kg) = leaf area/leaf dry weight; LDMC (g/g) = leaf dry weight/leaf fresh weight.

Functional traits vary between individuals due to intra- and inter-specific competition (Taylor et al., 2015). Thus, we used the community weighted mean trait (CWM_i_) to represent the average trait value of the forest.


(1) $$C\,W\,M_{\text{i}}=\frac{\sum_{\text{i}}^{\text{n}}D_{\text{i}}\times T\,r\,a\,i\,t}{\sum_{\text{i}}^{\text{n}}D_{\text{i}}}$$

where *CWM*_i_ represents the weighted characteristic values of the functional traits of the community and *D*_i_ represents the abundance of the dominant tree species. *Trait*_*i*_ represents the selected functional traits.

### Environmental data

The study found that plant resource utilization strategies are related to climate (Wang and Ali, 2021). Mean annual temperature (MAT), mean coldest monthly temperature (MCMT), mean warmest monthly temperature (MWMT), and (MAP) were extracted from the WorldClim global climate database at a spatial resolution of 1 km. Light is a key environmental factor affecting photosynthesis (Kramer, 1981), and we hypothesized that both annual sunshine duration (ASD) and mean annual evaporation (MAE) may be key predictors affecting plant resource utilization strategies. Both ASD and MAE were extracted from the Meteorological Data Center of the China Meteorological Administration.^1^

Soil conditions in different areas may potentially affect plant resource utilization strategies, so We extracted soil pH, soil N,^2^ and soil P^3^ data from a 250 m resolution grid in the top 30 cm soil level.

### Data analysis

The SLA and LDMC data we studied fit a continuous normal distribution. We used a significant difference test at the 0.05 significance level to test for significant differences between SLA and LDMC in the trees, shrubs, and herbs (Figure 2). Significant difference testing was performed using the R package *agricolae* (version 4.1.0, R Core Team, 2020).

**FIGURE 1:**
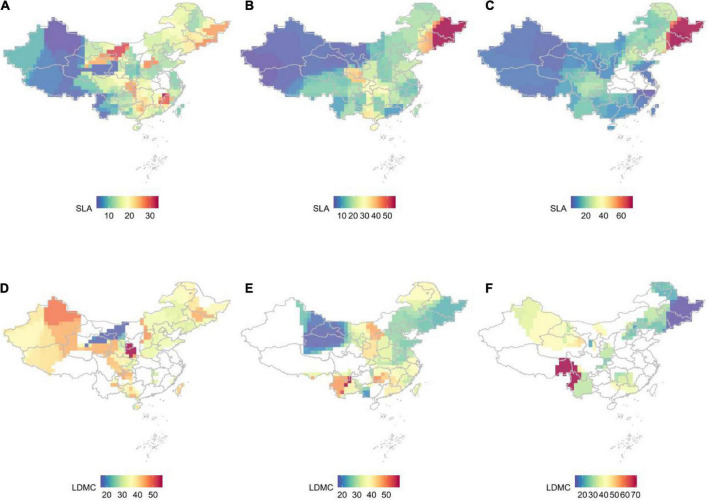
The distribution pattern of SLA and LDMC at different life forms in China, with a spatial resolution of 1 × 1 km, was studied by kernel density estimation. **(A)** SLA for trees; **(B)** SLA for shrubs; **(C)** SLA for herbs; **(D)** LDMC for trees; **(E)** LDMC for shrubs; **(F)** LDMC for herbs.

**FIGURE 2:**
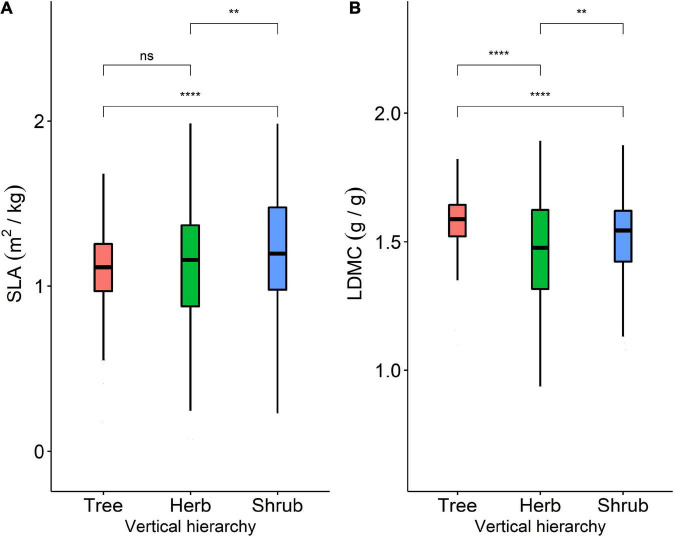
**(A)** Variability of SLA among trees, shrubs, and herbs; **(B)** variability of LDMC among trees, shrubs, and herbs. **Represents *P* < 0.01, ****represents *P* < 0.0001.

The contribution of environmental factors to spatial variation within SLA and LDMC was investigated using a linear regression model. *R*^2^ represents the goodness of fit of the model. Linear regression using the R package *lme4* (R Core Team, 2020).

Generalized additive models (GAMs) were used to evaluate the effects of various environmental factors on net primary productivity (NPP); this approach utilizes both parametric and non-parametric components to reduce model risks inherent to linear models (Zou et al., 2017). First, key environmental factors were selected using GAMs with a significance level of 0.05. Next, a single GAM was constructed to model how environmental factors were related to NPP; relationships were then visualized in a non-metric multidimensional scaling analysis (NMDS) (Sweeney et al., 2021). The model can be summarized as:


(2) $$\text{g}\,\left[E\,\left(Y|X\right)\right]=\sum_{\text{i}}\beta \,i\,X\,i+\sum_{\text{j}}f\,i\,\left(X\,i\right)+\varepsilon$$

where g(•) represents the link function, of which the specific form depends on the Y variable distribution. ϵ is the random error term; when a normal distribution function and identity-link are used, the errors are also normal. The link function is of the form g (u) = u, u = E (Y | X), E(ϵ | X) = 0. X_i_ is the explanatory variable, β_i_ is its corresponding parameter, and f_j_(•) is a non-parametric smoothing function corresponding to the explanatory variable X_j_. Here, a spline smoothing function S(•) (thin plate spline) was selected for function fitting between different nodes, and the penalty least square method was used to estimate each smoothing function S (•).

## Results

### Patterns of resource utilization strategies at different life forms in China

The resource utilization strategies of plants classified as trees, shrubs, and herbs show significant geographical differences but are highly consistent across similar latitude and longitude (Figure 1). As latitude and longitude increase, plant life strategy gradually changes from faster investment-return to slower investment-return, with SLA increasing from west to east and LDMC increasing from northeast to southwest at different verticals levels. Plants in the high mountains and high-altitude areas of the southwest tend to adopt a more conservative resource access strategy, while plants in the northeast obtain large amounts of available resources in the short term by improving SLA.

### Differences in utilization strategies of plant resources at different life forms

The SLA and LDMC of plants at different life forms show significant differences (*, *P* < 0.05). Among the three life forms, shrub SLA and tree LDMC were higher (Figure 2). Moving across vegetation type from tree to shrub to herb, resource utilization strategies vary, gradually shifting from a slower (i.e., conservative) to a faster investment-return strategy. Shrub SLA was significantly higher than herb SLA, which was significantly higher than tree LDMC (Figure 2A). Tree LDMC was significantly higher than that of herbs and shrubs (Figure 2B).

### Effects of environmental factors on plant resource utilization strategies at different life forms

**FIGURE 3:**
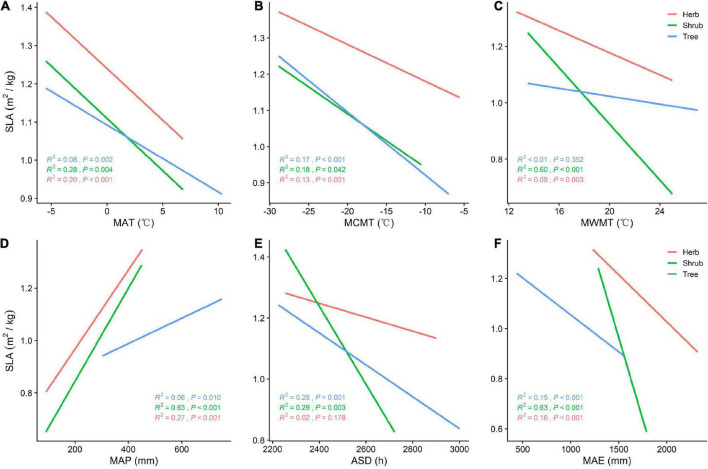
**(A)** General linear relationship between MAT and SLA of plants in the trees, shrubs, and herbs; **(B)** general linear relationship between MCMT and SLA of plants in the trees, shrubs, and herbs; **(C)** general linear relationship between MWMT and SLA of plants in the trees, shrubs, and herbs; **(D)** general linear relationship between MAP and SLA of plants in the trees, shrubs, and herbs; **(E)** general linear relationship between ASD and SLA of plants in the trees, shrubs, and herbs; **(F)** general linear relationship between MAE and SLA of plants in the trees, shrubs, and herbs.

**FIGURE 4:**
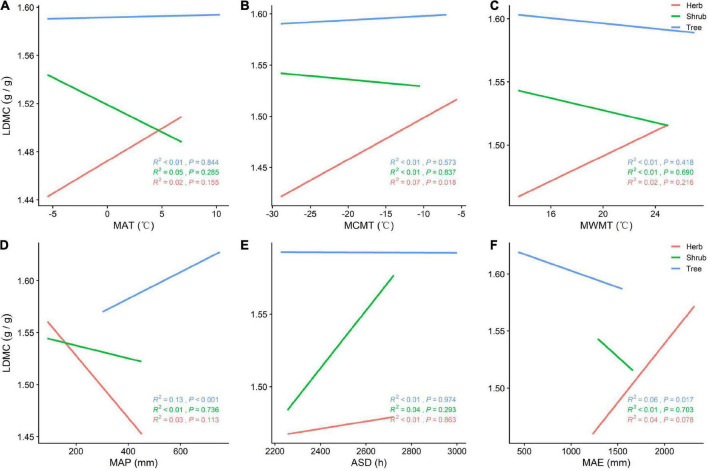
**(A)** General linear relationship between MAT and LDMC of plants in the trees, shrubs, and herbs; **(B)** general linear relationship between MCMT and LDMC of plants in the trees, shrubs, and herbs; **(C)** general linear relationship between MWMT and LDMC of plants in the trees, shrubs, and herbs; **(D)** general linear relationship between MAP and LDMC of plants in the trees, shrubs, and herbs; **(E)** general linear relationship between ASD and LDMC of plants in the trees, shrubs, and herbs; **(F)** general linear relationship between MAE and LDMC of plants in the trees, shrubs, and herbs.

SLA is positively correlated with MAP (*P* < 0.05) and negatively correlated with MAT, MCMT, ASD, and MAE across all vegetation types (Figures 3A–F). With increasing MAP, both SLA and tree LDMC increases, while SLA increases and LDMC decreases among herbs and shrubs (Figures 3D, 4D). The LDMC of herbs and shrubs increased significantly with increasing ASD (Figure 4E) and tree LDMC decreased significantly with increasing MAE (Figures 4D,F). Under better hydrothermal conditions, plants across life forms shift to a faster investment-return survival strategy, while plants exhibit a more conservative survival strategy as MAE and MAT increase. Of all the climatic factors, (Figure 3A–F, Figure 4A–F) ASD predicted SLA better for the trees and shrubs (*R*^2^ = 0.25, 0.29, *P* < 0.001; Figure 3E) and MAP predicted LDMC best for the trees (*R*^2^ = 0.13, *P* < 0.001; Figure 4D).

**FIGURE 5:**
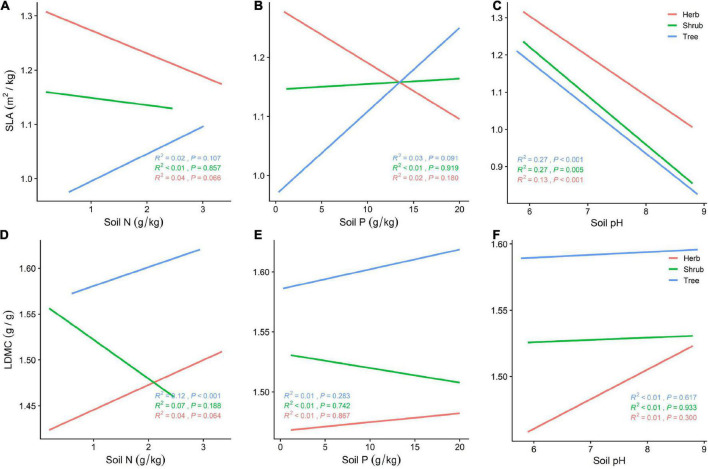
**(A)** General linear relationship between Soil N and SLA of plants in the trees, shrubs, and herbs; **(B)** general linear relationship between Soil P and SLA of plants in the trees, shrubs, and herbs; **(C)** general linear relationship between Soil pH and SLA of plants in the trees, shrubs, and herbs; **(D)** general linear relationship between Soil N and LDMC of plants in the trees, shrubs, and herbs; **(E)** general linear relationship between Soil P and LDMC of plants in the trees, shrubs, and herbs; **(F)** general linear relationship between Soil pH and LDMC of plants in the trees, shrubs, and herbs.

SLA is negatively correlated with soil pH at different life forms (Figure 5C), while LDMC increases with soil pH, with plants adopting a more conservative survival strategy. Tree and herb SLA and LDMC increase with increasing soil N and P, while shrubs exhibit increased SLA and decreased LDMC (Figures 5A,B,D,E). With increasing soil nutrients, plants switch to a faster investment-return survival strategy. Of all the soil nutrient factors, SLA was best predicted by soil pH for shrubs and trees (*R*^2^ = 0.27, *P* < 0.001; Figure 5A–F).

### Effects of environmental factors on changes in plant resource utilization strategies in different communities

**FIGURE 6:**
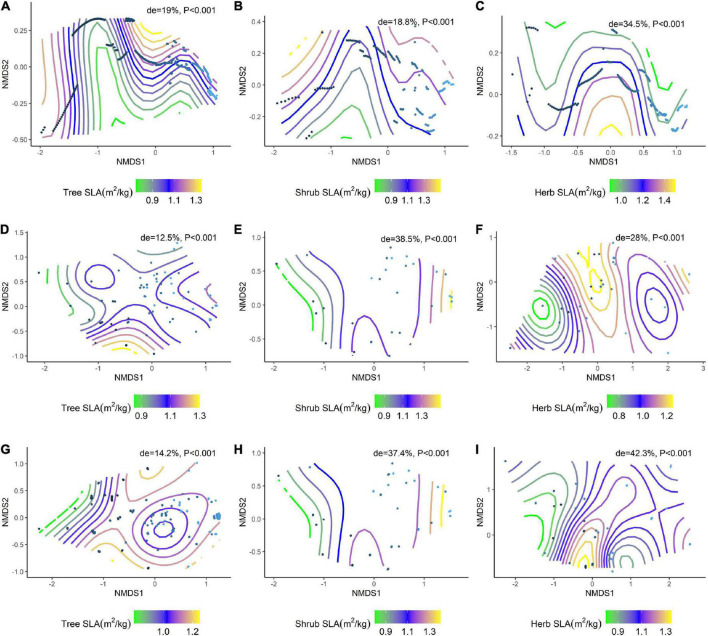
NMDS ranking of climatic and soil factors with different life forms of SLA. **(A)** NMDS ranking of soil factors with tree lSLA; **(B)** NMDS ranking of soil factors with shrub SLA; **(C)** NMDS ranking of soil factors with herb SLA; **(D)** NMDS ranking of climatic factors with tree SLA; **(E)** NMDS ranking of climatic factors with shrub SLA; **(F)** NMDS ranking of climate factors and herb SLA; **(G)** NMDS ranking of the sum of soil factors and climate factors and tree SLA; **(H)** NMDS ranking of the sum of soil factors and climate factors and shrub SLA; **(I)** NMDS ranking of the sum of soil factors and climate factors and herb SLA. Trait stacking indicates that abiotic factors, indicated by points on the NMD, are associated with higher or lower trait values, consistent with a colored trait gradient. Note that if the relationship between SLA and abiotic factors is linear, the gradient splines are parallel. Non-linear relationships between SLA and abiotic factors are represented by curve splines.

**FIGURE 7:**
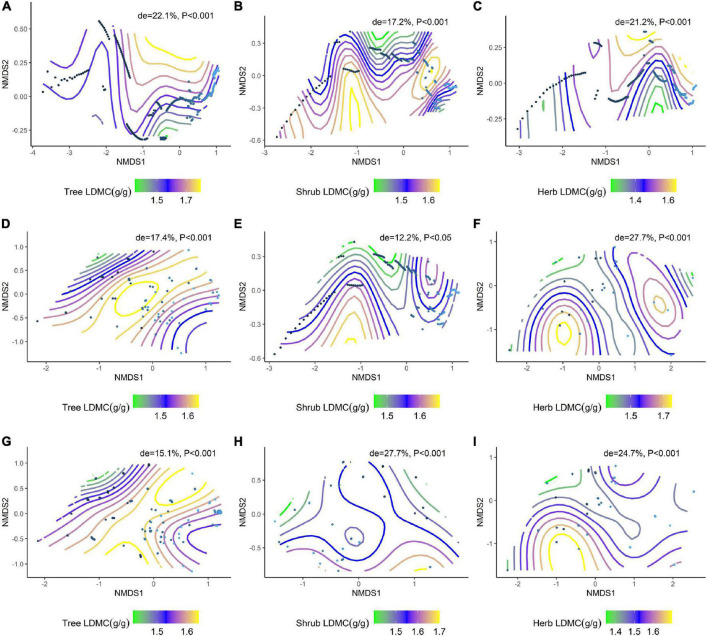
NMDS ranking of climatic and soil factors with different life forms of LDMC. **(A)** NMDS ranking of soil factors with tree LDMC; **(B)** NMDS ranking of soil factors with shrub LDMC; **(C)** NMDS ranking of soil factors with herb LDMC; **(D)** NMDS ranking of climatic factors with tree LDMC; **(E)** NMDS ranking of climatic factors with shrub LDMC; **(F)** NMDS ranking of climate factors and herb LDMC; **(G)** NMDS ranking of the sum of soil factors and climate factors and tree LDMC; **(H)** NMDS ranking of the sum of soil factors and climate factors and shrub LDMC; **(I)** NMDS ranking of the sum of soil factors and climate factors and herb LDMC. Trait stacking indicates that abiotic factors, indicated by points on the NMD, are associated with higher or lower trait values, consistent with a colored trait gradient. Note that if the relationship between LDMC and abiotic factors is linear, the gradient splines will be parallel. Non-linear relationships between LDMC and abiotic factors are represented by curve splines.

We further analyzed the effects of environmental factors on SLA and LDMC at different life forms based on a generalized additive model (GAM) with non-metric multidimensional scaling (NMDS) ranking (Figure 6A–I; Figure 7A–I). Soil nutrient factors explained the variation in resource utilization strategies of plants at the tree level (Figure 6A, de = 19.0%; Figure 7A, de = 22.1%), while climatic factors best explained herb and shrub strategies (Figures 6E,F, de = 38.5%, 28%; Figures 7E,F, de = 12.2%, 27.7%). Climate and soil nutrient factors jointly shape the variation in SLA and LDMC of plants at different life forms.

## Discussion

Significant differences exist among plant resource utilization strategies at different life forms in the forest community (Li Y. et al., 2021). SLA of understory plants is significantly higher than that of canopy plants and LDMC is significantly higher in canopy plants than in understory plants (Treml et al., 2019). There is a significant negative correlation between SLA and LDMC (Worthy et al., 2020). Shrubs and herbs, which are affected by the shading of tree foliage, adapt to low light conditions by increasing leaf area and SLA to capture more light energy, adopting a faster investment-return resource utilization strategy (Rüger et al., 2012).

There is a trade-off between leaf lifespan and photosynthetic productivity in plants (Yin et al., 2018). Understory plants increase photosynthetic efficiency at the expense of leaf lifespan, increasing SLA and reducing LDMC as the dry matter content of leaves decreases (Kröber et al., 2015). Due to the differences between species, herb plants are more adapted to understory shady environments, and the increase in SLA will be less than that of shrubs (Ma et al., 2021). Though tree leaves intercept light energy efficiently, their stomata close because of continuous and intense solar radiation. To reduce water loss due to transpiration, vegetation belonging to this life form enhance the amount of dry matter contained in their leaves (Baribault et al., 2010). Thus, the slower investment-return resource utilization strategy of tree leaves results in higher LDMC.

Habitat heterogeneity significantly affects the resource utilization strategy of plants (Guimarães et al., 2020). The SLA of plants at different life forms increases significantly with increasing longitude, while the LDMC increases with increasing latitude. From north to south and from west to east, precipitation and temperature increase (Wu et al., 2019). When plants experience water stress due to reduced precipitation, they close their stomata, reduce SLA, and compact their mesophyll cells to reduce water diffusion. These strategies enhance the amount of water retained in leaves, and high plant resource retention represents high plant survival (Galmés et al., 2013). The SLA of plant communities was significantly positively correlated with precipitation (Anderson et al., 2020). Similarly, temperature is an important factor in the spatial and temporal distribution of plant resource utilization strategies, as it directly affects the activity of enzymes associated with photosynthesis and respiration (Descombes et al., 2020). Previous research has shown that, under low temperatures, plants adopt a slower investment-return strategy, reducing SLA and increasing LDMC to devote more dry matter to improving freezing tolerance. Similarly, under conditions high temperature and intense solar radiation, plants must increase dry matter and reduce SLA to reduce the photosynthetic rate and prevent water evaporation (Coble and Cavaleri, 2015). Trees in China are concentrated in southern regions where precipitation is high, and shrubs and herbs are concentrated in arid, semi-arid, and alpine high-altitude mountainous areas in the northwest (Zhao and Wu, 2014). This distribution explains the increasing trend of SLA and LDMC with increasing latitude and longitude (Tian et al., 2016). Herb and shrub LDMC decreases with MAP and in herb and shrub levels increases with ASD because understory growth is limited by the amount of light penetrating the canopy. Higher ASD raises ambient forest temperatures, but it also increases the amount of light energy reaching the understory, enhancing rates of photosynthesis. As ASD increases, so does LDMC as foliage accumulates dry matter, strengthening leaf structures (e.g., cell walls and microtubules) and prolonging leaf lifespan (Cheng et al., 2016). Excessive rainfall decreases soil conductivity and photosynthetically effective radiation, and understory herbs and shrubs in low light and rainy conditions tend to increase SLA and decrease LDMC to maximize resource access (Kröber et al., 2015). Therefore, at the community level, hydrothermal conditions (especially MAP) are the principal factors driving plant resource utilization strategies (He et al., 2010).

Soil provides most of the nutrients required for plant growth, and soil nutrients are closely related to plant leaf resource utilization strategies (Gao et al., 2019). N and P are nutrients that are not only important for photosynthesis but that are also components of biomolecules such as nucleic acids and proteins (Yang et al., 2017). Soil N and P content directly affect plant leaf N (LN) and leaf P (LP) content, and LN and LP contribute to plant productivity and photosynthesis. Higher LN enhances the efficiency of carbon dioxide (CO_2_) and N absorption, and higher LN is strongly correlated with improved stomatal conductance (Goedhart et al., 2010). The higher SLA of plants growing in high nutrient environments facilitates rapid access to soil resources; thus SLA is positively correlated with soil N and P content and negatively correlated with soil pH (Hodgson et al., 2011). This is consistent with our findings that tree, shrub, and herb SLA increases with increasing soil N and P content and decreases with increasing soil pH. The LDMC of plants in different life forms also increases with increasing soil N and P but, unlike SLA, LDMC also increases with soil pH. Other experiments have also shown that SLA, LN, and LP are lower and LDMC is higher in green leaves at higher pH (Tao et al., 2019), lending further support to the claim that plants growing in acidic conditions exhibit a slower investment-return survival strategy. Soil organic matter content is significantly and positively correlated with the resource utilization strategies of plants at different life forms (Niu et al., 2020).

Plant resource utilization strategies are influenced by environmental conditions, with temperature, precipitation, and soil nutrients acting together to influence plant leaf traits (Goll et al., 2017). Generalized additive model analysis shows that SLA and LDMC across all life forms are mainly affected by soil nutrient factors, with climatic factors playing a key role in shaping the pattern of SLA and LDMC in the shrub and herb levels. Plants with higher SLA have a faster investment-return survival strategy, with efficient water and nutrient utilization supporting rapid growth (Pietsch et al., 2014). Higher LDMC in plants is associated with reduced leaf water holding capacity (Yu et al., 2009) and slower growth. Soil nutrient status, lower nutrient uptake and leaf water holding capacity, and low SLA and high LDMC of vegetation’s topmost foliage is particularly critical for trees compared to shrubs and herbs factors on trees are particularly critical. In contrast, shrubs and herbs are more affected by light vs. shade.

Across vegetation types, differences in SLA and LDMC are primarily driven by hydrothermal conditions. Precipitation can accelerate the leaching and transformation of soil nutrients, while temperature provides a suitable environment for soil microorganisms to better decompose soil organic matter (Gao et al., 2021). Studies have found that soil nutrients are the main factors affecting plant resource utilization strategies, while the availability of soil nutrients depends on climatic factors and soil microorganisms (Jiao, 2021). Climate can directly act on soil nutrients and dominate plant resource utilization strategies. The significant differences in plant resource utilization strategies at different life forms of the community imply that plants have different growth strategies in response to different environmental factors (Madani et al., 2017). Thus, soil nutrient and climate factors combine to affect the distribution of resource utilization strategies associated with trees, shrubs, and herbs, and the contribution of climatic factors was greater than that of soil nutrient factors.

## Conclusion

We used 926 forest plots from 163 sites in China surveyed between 2005 and 2020 to verify the driving effects of climatic and soil nutrient factors on forest resource utilization strategies. Our results show significant differences in the resource utilization strategies of plants classified as trees, shrubs, and herbs. Plants in the herb and shrub layers will mostly adopt a faster investment-return strategy, while plants at the tree level will choose to adapt to their environment in a more conservative way. MAP is a key factor in shaping the spatial pattern of resource utilization strategies of plants at different life forms. Both climate and soil significantly affect plant resource access and utilization strategies, but the relative contribution of climate factors is greater than that of soil nutrient factors. In the context of global environmental change, this study provides a theoretical framework for understanding the resource utilization strategies of plants.

## Data availability statement

The original contributions presented in this study are included in the article/Supplementary material, further inquiries can be directed to the corresponding author.

## Author contributions

JG: conceptualization, experiment implementation, visualization, and validation. XW and JW: formal analysis. XW: writing—original draft preparation. XW, JW, YJ, and JG: writing—review and editing. JG and YJ: project administration and funding acquisition. All authors have read and agreed to the published version of the manuscript.

## Footnotes

1
https://data.cma.cn/

2
http://www.csdn.store

3
https://www.osgeo.cn/data/wc137
