# Supplementary figures and images for: Climate factors determine the utilization strategy of forest plant resources at large scales
Source: Front Plant Sci. 2022 Aug 10;13:990441. doi: 10.3389/fpls.2022.990441 (PMC9399733; doi:10.3389/fpls.2022.990441)

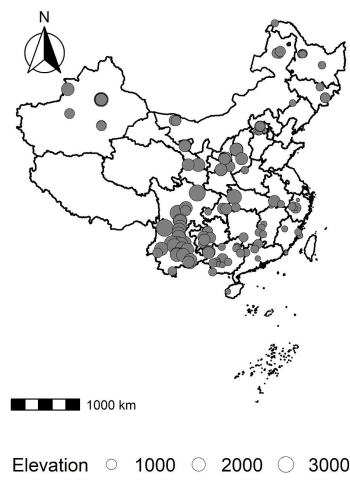

**FIGURE S1** | The geographical location of forest plots in this study.

Supplement: Supplementary file 1 [file Presentation_1.pdf]
